# Tissue tropism of toxic metals in northern quolls (*Dasyurus hallucatus*) and northern brown bandicoots (*Isoodon macrourus*) on Groote Eylandt, Australia

**DOI:** 10.1371/journal.pone.0322386

**Published:** 2025-06-25

**Authors:** Elise M. Contreras, Frank A. von Hippel, Skye Cameron, Kaylah del Simone, Ami F. Amir Abdul Nasir, John Postlethwait, Robbie Wilson

**Affiliations:** 1 Department of Biological Sciences, Northern Arizona University, Flagstaff, Arizona, United States of America; 2 Department of Community, Environment & Policy, University of Arizona, Tucson, Arizona, United States of America; 3 School of The Environment, The University of Queensland, St Lucia, Queensland, Australia; 4 Institute of Neuroscience, University of Oregon, Eugene, Oregon, United States of America; Namik Kemal University: Tekirdag Namik Kemal Universitesi, TÜRKIYE

## Abstract

Mining is an essential part of the Australian economy, but can create environmental concerns due to toxic metal pollution. Surrounding active manganese (Mn) mining sites, such as those on Groote Eylandt, Australia, toxic metal exposure leads to variation in the internal distribution within animals (i.e., tissue tropism) and can exert long-term health effects on wildlife. We aimed to determine if hair of the endangered northern quoll (*Dasyurus hallucatus*) or of the northern brown bandicoot (*Isoodon macrourus*) would be sufficient to monitor internal contamination. We analyzed nine toxic metals (Al, Cd, Co, Cr, Cu, Mn, Ni, Pb, Zn) in eight tissues/organs (cerebellum, hair, kidney, liver, lung, neocortex, olfactory bulb, testes) of quolls and bandicoots using inductively coupled plasma – optical emission spectroscopy (ICP-OES). We found six significant positive and five significant negative correlations between the concentration of metals in internal tissues and the concentration in hair in quolls, and four significant relationships in bandicoots, all negative. We also found that the concentrations of metals in quoll tissues/organs, except for hair, were significantly higher than in bandicoots. Differences in the magnitude and direction of these relationships may reflect differences in life histories or metabolic rates. The concentration of Mn in hair was significantly higher in quolls collected near the mining sites than in quolls collected at distant locations, and this also appeared to be the case for bandicoots, but we lacked a sufficient sample size to demonstrate this statistically. The concentration of Al in the hair of quolls was also significantly higher near the mining sites. The concentration of Mn in the hair of quolls reflected the concentration of Mn in the cerebellum and neocortex, while the concentration of Al in the hair of quolls reflected Al concentration in the cerebellum, neocortex, liver, and kidney. We conclude that hair analyzed with ICP-OES is an effective biomarker of local exposure to Mn and Al for quolls, and that hair Mn and Al concentration in quolls can be used as a biomarker of concentration of some tissues, such as cerebellum and neocortex. These findings point to hair as a valuable non-invasive method for assessing metal exposure in wildlife that can be useful for management and conservation efforts.

## Introduction

Manganese (Mn) is the fifth most abundant metal in the environment and is an important component in the production of steel, certain pesticides, and dry cell batteries [1]. In animals, Mn is also an essential trace element that is required for proper immune function, bone growth, and cellular metabolism [2]. Although small amounts of dietary Mn are vital for proper bodily function, chronic exposure to high concentrations of Mn causes progressive neurological damage that impacts cognitive and motor systems [1,3–5]. Inhalation of Mn particles is the primary mode of toxicity [6,7] making Mn dust a health concern surrounding open mining sites. Fine dust particles (<2.5um) can be carried long distances, impacting local ecosystems [8,9].

When inhaled, Mn is deposited throughout the respiratory tract, including lung alveoli, and passes into the bloodstream [3,5,10–12]. It also accumulates in the brain via passage from the olfactory nerve [10]. Although the brain is the site of greatest accumulation, Mn accumulates in other non-brain tissues and organs as well [7,13,14]. Respirable Mn levels around active mining sites often exceed international recommendations (0.05ug/m^3^) [15]; for example, exceedances are found as far as 20 km from the active mining and storage sites on Groote Eylandt, Australia [8].

Groote Eylandt is an Indigenous Protected Area of international conservation significance [16,17]. The island protects 12 threatened and endangered species including the northern quoll (*Dasyurus hallucatus*), a semi-arboreal carnivore [18]. Despite their endangered status in mainland Australia, the northern quoll maintains a healthy population size on Groote Eylandt [16,17]. Similarly, other species experiencing declines in at least part of their range, such as the insectivorous northern brown bandicoot (*Isoodon macrourus*) [19], have robust populations on Groote Eylandt.

Mining activities on Groote Eylandt pose a potential conservation threat to local wildlife. High concentrations of toxic metals (e.g., cadmium [Cd], copper [Cu], chromium [Cr], Mn, lead [Pb], zinc [Zn]) are often found in soil surrounding Mn contaminated sites [20]. Due to their persistence in the environment, toxic metals can exert long-term health effects on local wildlife [21]. We found that northern quolls living near the Groote Eylandt mine accumulated Mn in the cerebellum, neocortex, testes, and hair at higher levels than did quolls living far from the mine [8]. Increased Mn body burden in quolls was associated with reduced running speed approaching a turn, which may impact survival [22]. Even at sub-lethal levels, toxins may affect survival and reproduction by increasing predation risk, decreasing competitive abilities, or reducing the number of viable offspring [23,24].

In this study, we analyzed nine toxic metals (aluminum [Al], Cd, cobalt [Co], Cr, Cu, Mn, nickel [Ni], Pb, Zn) in eight tissues (cerebellum, hair, kidney, liver, lung, neocortex, olfactory bulb, testes) in quolls and bandicoots. We compared quolls and bandicoots because they represent different feeding guilds on the island, and hence allow comparisons across trophic ecology. Foo et al. [25] found that human hair could be used to indicate environmental exposure to Mn. As a less invasive sampling approach, we aimed to determine if hair would similarly be sufficient to monitor internal contamination in quolls and bandicoots. We hypothesized that the hair of both species would be an effective biomarker of exposure to Mn from mining sites (higher Mn concentrations in the hair of animals near the mine than at distant locations) and that concentrations of Mn and other toxic metals in hair would be significantly correlated with internal tissue concentrations.

## Methods

### Animal trapping and tissue sampling

Male northern quolls and male northern brown bandicoots were collected over two field seasons: quolls in August-October 2015 and bandicoots in August 2018. Semelparity of the male quoll results in the male mating with as many females as possible during a three-week period and then dying shortly afterward [26]. To avoid impacting the population, we collected the male quolls during the brief period after all sampled females in the study area were impregnated (verified through pouch inspection) and before the males died naturally. Similarly, although male bandicoots are not semelparous, we only collected males to minimize impacts on the population. We aimed to collect twenty males of each species (ten from near the mine and ten from a distant location), as specified in our permit. However, we only succeeded in trapping 18 quolls and nine bandicoots.

Sherman traps were set in the evening in locations near and far from the mine, baited with canned dog food, and left overnight. Traps were checked early the next morning. Captured males were transported in cloth bags to the Anindilyakwa Threatened Species Center for processing. All quolls were sampled at one year of age. Bandicoot individuals varied in age, being either one or two years old, based on assessment of tooth wear [26,27].

The location of capture, morphometric data, and age were all documented. Roughly half of the animals were collected from sites adjacent to mining activities (hereafter referred to as high exposure individuals on the assumption that proximity relates to exposure) and half were collected from distant locations (hereafter referred to as low exposure individuals). Upon completion of measurements, individuals were gently restrained in the cloth bag and euthanized via decapitation using a commercially available small-animal guillotine (Kent Scientific, Connecticut, USA). This is an approved method of euthanasia due to the rapidity of application with little apparent stress [28]. All research methods were approved by the University of Queensland animal ethics committee (permit numbers SBS/541/12/ANINDILYAKWA/MYBRAINSC and BS/010/16/ARC) and the Northern Territory Parks and Wildlife Commission (permit numbers 47603 and 62497). Immediately upon euthanasia, target tissues were dissected from the right side of the body and stored at −80°C. Hair was plucked from between the shoulder blades and stored at room temperature.

### Metals analysis

Hair samples were washed with deionized water mixed thoroughly with 1% Triton® X100 non-ionic detergent solution, and rinsed in deionized water at least 8x to remove detergent residues. Each sample was then dried in an oven at 110°C for an hour and weighed to estimate element concentrations per-kg of dried sample. This detergent-based hair washing procedure was adapted from Filistowicz et al. [29] and Kempson and Skinner [30]. Hair samples were then mixed with nitric acid and hydrochloric acid solution (3:1 ratio) and closed-vessel digested using an Ethos 1 microwave digester (Milestone, Sorisole, Italy), diluted in triple deionized water, and analyzed by inductively coupled plasma – optical emission spectroscopy (ICP-OES; Varian, Santa Clara, California, USA). Tissue samples were digested and analyzed similar to the hair samples, except with a nitric acid and perchloric acid solution (5:1 ratio). Nine metals were analyzed (Al, Cd, Co, Cr, Cu, Mn, Ni, Pb, Zn) in eight tissues/organs (cerebellum, hair, kidney, liver, lung, neocortex, olfactory bulb, testes). Kidney was analyzed only in quolls.

### Statistical analysis

Statistical analyses were completed using R version 4.2.1. Approximately half of the animals for each species were collected from sites close to mining activities (quoll n = 8, bandicoot n = 5) and half from distant locations (quoll n = 10, bandicoot n = 4). Due to the small sample sizes, we used nonparametric statistics. Quolls and bandicoots were modeled separately for all tissues and organs using Spearman’s rank correlation. Note that for comparisons in Mn concentration between hair and other tissues/organs in quolls, this paper presents a re-analysis of data from [8], which only examined quolls, and did not compare hair and internal tissues/organs for other metals. Mann-Whitney U tests were also performed to test for differences between quolls and bandicoots for all tissues/organs.

## Results

We hypothesized that the hair of both quolls and bandicoots would be an effective biomarker of exposure to Mn from mining sites, with the expectation of higher Mn concentrations in the hair of animals near the mine than at distant locations. Given that other toxic metals often co-occur at Mn mining sites, hair might also reflect exposure to these additional elements.

**Fig 1:**
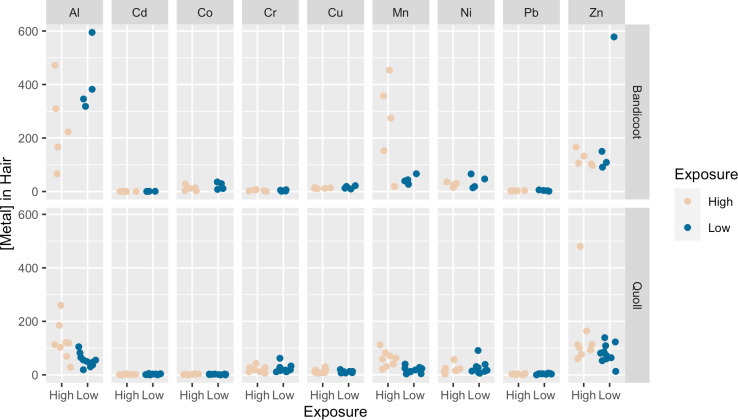
Al and Mn levels in hair of quolls showed a significant difference in hair between individuals living near the mine than far from the mine.

Quolls showed higher concentrations of Mn (U = 74, p = 0.002) and Al (U = 68, p = 0.01) in the hair of individuals collected near the mine than far from it. Otherwise, high and low exposure individuals did not differ in toxic metal content of hair for either species (Fig 1; see S1 Data for all results).

We also hypothesized that concentrations of Mn and other toxic metals in hair would be significantly correlated with internal tissue concentrations. To test this, we examined relationships between concentrations in hair and those in the cerebellum, kidney, liver, lung, neocortex, olfactory bulb, and testes.

### Cerebellum

**Fig 2:**
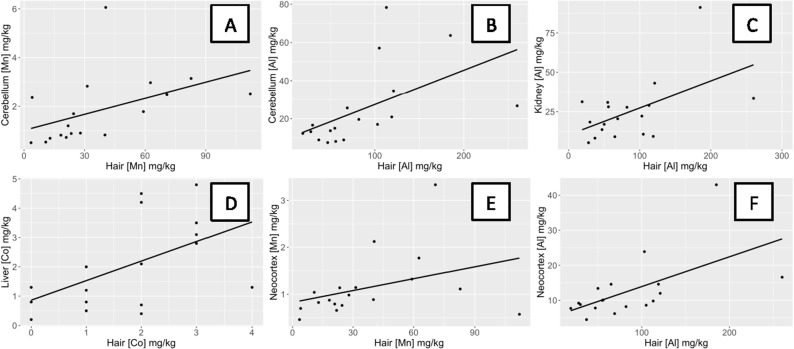
Positive relationships between concentrations of metals in the hair and internal tissues of quolls. Quolls showed a significant positive relationship between the concentration of (A) Mn in hair and in the cerebellum (ρ = 0.76, p < 0.001), (B) Al in hair and in the cerebellum (ρ = 0.77, p < 0.001), (C) Al in hair and in the kidney (ρ = 0.45, p = 0.05), (D) Co in hair and in the liver (ρ = 0.57, p = 0.01), (E) Mn in hair and in the neocortex (ρ = 0.52, p = 0.02), and (F) Al in hair and in the neocortex (ρ = 0.60, p = 0.007).

Quolls showed a significant positive relationship between metal concentrations in hair and in the cerebellum for Al (ρ = 0.76, p < 0.001) and Mn (ρ = 0.76, p < 0.001) (Table 1; Fig 2). For all remaining metals, no relationship was observed between the concentration in hair and the concentration in the cerebellum (Table 1).

**Table 1:** The Spearman’s rank correlation coefficient (ρ) and the significance (p) between the concentration of metals in hair and in each target tissue for quolls.

	Al	Cd	Co	Cr	Cu	Mn	Ni	Pb	Zn
**Cerebellum**	**π = 0.764** **p < 0.001**	π = 0.067 p = 0.79	π = −0.204 p = 0.41	π = −0.200 p = 0.42	π = 0.238 p = 0.33	**π = 0.764** **p < 0.001**	π = 0.021 p = 0.93	π = 0.143 p = 0.56	π = 0.306 p = 0.21
**Kidney**	**π = 0.457** **p = 0.05**	π = 0.176 p = 0.48	π = −0.191 p = 0.44	π = 0.152 p = 0.54	π = 0.393 p = 0.10	π = −0.048 p = 0.84	π = −0.010 p = 0.96	π = 0.383 p = 0.11	**π = −0.536** **p = 0.02**
**Liver**	**π = −0.457** **p = 0.05**	π = −0.064 p = 0.79	**π = 0.571** **p = 0.01**	π = 0.336 p = 0.17	π = 0.271 p = 0.27	π = 0.205 p = 0.41	π = 0.142 p = 0.57	π = 0.15 p = 0.70	**π = −0.536** **p = 0.02**
**Lung**	π = 0.051 p = 0.84	π = −0.154 p = 0.55	π = −0.358 p = 0.15	π = −0.104 p = 0.68	**π = −0.621** **p = 0.007**	π = −0.139 p = 0.58	π = −0.170 p = 0.51	π = 0.174 p = 0.50	π = −0.007 p = 0.97
**Neocortex**	**π = 0.604** **p = 0.007**	π = −0.060 p = 0.81	**π = −0.532** **p = 0.02**	π = −0.148 p = 0.55	π = 0.140 p = 0.57	**π = 0.521** **p = 0.02**	π = −0.299 p = 0.22	π = 0.145 p = 0.56	π = 0.127 p = 0.61
**Olfactory Bulb**	π = −0.044 p = 0.86	π = 0.123 p = 0.62	π = −0.271 p = 0.27	π = −0.275 p = 0.26	π = −0.053 p = 0.83	π = −0.306 p = 0.21	π = 0.267 p = 0.28	π = 0.153 p = 0.54	π = 0.121 p = 0.63
**Testes**	π = 0.067 p = 0.79	π = −0.304 p = 0.21	π = 0.060 p = 0.81	π = 0.008 p = 0.97	π = 0.345 p = 0.16	π = 0.223 p = 0.37	π = −0.395 p = 0.10	π = 0.410 p = 0.09	π = −0.322 p = 0.19

**Fig 3:**
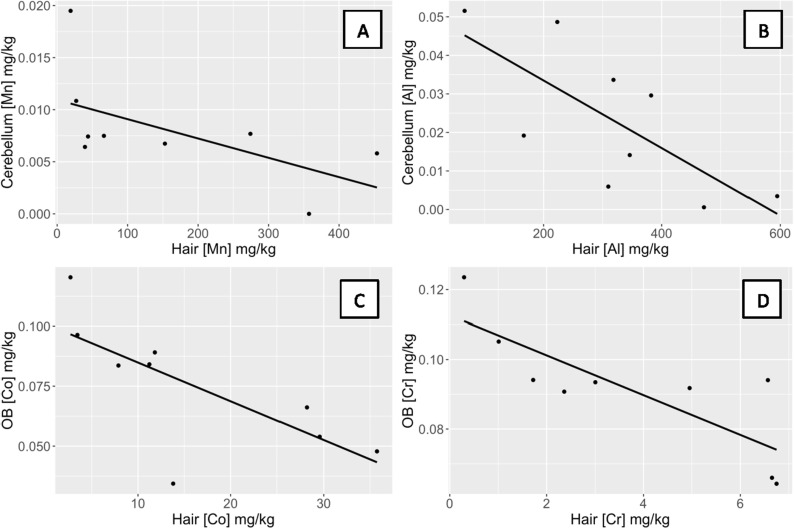
Negative relationships between concentrations of metals in the hair and internal tissues of bandicoots. Bandicoots showed a significant negative relationship between the concentration of (A) Mn in hair and in the cerebellum (ρ = −0.70, p = 0.04), (B) Al in hair and in the cerebellum (ρ = −0.71, p = 0.03), (C) Co in hair and in the olfactory bulb (ρ = −0.83, p = 0.008), and (D) Cr in hair and in the olfactory bulb (ρ = −0.85, p = 0.006).

Bandicoots showed a significant negative relationship between metal concentrations in hair and in the cerebellum for Al (ρ = −0.71, p = 0.03) and Mn (ρ = −0.70, p = 0.04) (Table 2; Fig 3). For all remaining metals, no relationship was observed between the concentration in hair and the concentration in the cerebellum (Table 2). Quolls and bandicoots showed a significant difference between each other in tissue concentrations for all metals in the cerebellum, with quolls consistently showing higher concentrations than bandicoots (p < 0.001).

**Table 2:** The Spearman’s rank correlation coefficient (ρ) and the significance (p) between the concentration of metals in hair and in each target tissue for bandicoots.

	Al	Cd	Co	Cr	Cu	Mn	Ni	Pb	Zn
**Cerebellum**	**π = −0.716** **p = 0.03**	π = 0.067 p = 0.79	π = 0.183 p = 0.64	π = −0.4 p = 0.29	π = −0.4 p = 0.29	**π = −0.7** **p = 0.04**	π = −0.133 p = 0.74	π = 0.15 p = 0.70	π = −0.45 p = 0.22
**Liver**	π = −0.583 p = 0.10	π = 0.4 p = 0.29	π = −0.166 p = 0.67	π = 0.116 p = 0.77	π = −0.65 p = 0.06	π = 0.133 p = 0.74	π = −0.066 p = 0.88	π = 0.15 p = 0.70	π = −0.45 p = 0.22
**Lung**	π = 0.416 p = 0.26	π = −0.05 p = 0.91	π = 0.016 p = 0.98	π = −0.283 p = 0.46	π = −0.466 p = 0.21	π = 0.351 p = 0.35	π = −0.333 p = 0.38	π = 0.266 p = 0.49	π = −0.266 p = 0.49
**Neocortex**	π = 0.051 p = 0.84	π = −0.366 p = 0.33	π = 0.166 p = 0.67	π = −0.3 p = 0.43	π = 0.583 p = 0.10	π = −0.355 p = 0.34	π = 0.1 p = 0.81	π = 0.266 p = 0.49	π = 0.516 p = 0.16
**Olfactory Bulb**	π = 0.483 p = 0.19	π = −0.033 p = 0.94	**π = −0.833** **p = 0.008**	**π = −0.85** **p = 0.006**	π = 0.466 p = 0.21	π = −0.516 p = 0.16	π = 0.1 p = 0.81	π = 0.2 p = 0.61	π = 0.166 p = 0.67
**Testes**	π = −0.5 p = 0.17	π = 0.45 p = 0.22	π = −0.316 p = 0.41	π = −0.416 p = 0.26	π = 0.216 p = 0.58	π = 0.35 p = 0.35	π = 0.383 p = 0.31	π = 0.35 p = 0.35	π = 0.066 p = 0.88

### Kidney

**Fig 4:**
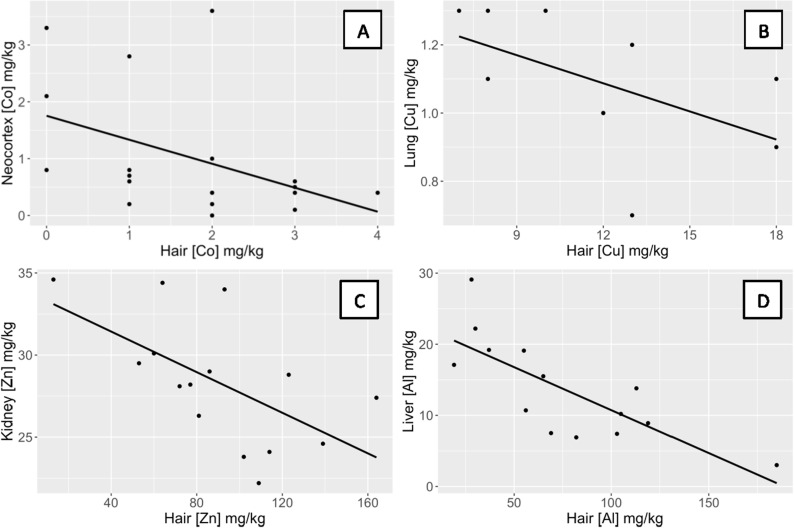
Negative relationships between concentrations of metals in the hair and internal tissues of quolls. Quolls showed a significant negative relationship between the concentration of (A) Co in hair and in the neocortex (ρ = −0.53, p = 0.02), (B) Cu in hair and in the lung (ρ = −0.64, p < 0.04), (C) Zn in hair and in the kidney (ρ = −0.66, p = 0.008), and (D) Al in hair and in the liver (ρ = −0.79, p = 0.001). Outliers were removed. The figures with outliers included are shown in S1 Fig.

Quolls showed a significant relationship between the concentration of metals in hair and the concentration in the kidney for Al (ρ = 0.45, p = 0.05; Fig 2) and Zn (ρ = −0.53, p = 0.02; Fig 4). For all remaining metals, no relationship was observed between the concentration in hair and the concentration in the kidney (Table 1). The kidney was not examined in bandicoots.

### Liver

Quolls showed a significant relationship between the concentration of metals in hair and the concentration in the liver for Al (ρ = −0.45, p = 0.05; Fig 4), Co (ρ = 0.57, p = 0.01; Fig 2), and Zn (ρ = −0.53, p = 0.02; Fig 4). For all remaining metals, no relationship was observed between the concentration in hair and the concentration in the liver (Table 1).

Bandicoots showed no significant relationships between the concentration of metals in hair and the concentration in the liver (Table 2). Quolls and bandicoots showed a significant difference between each other in tissue concentrations for all metals in the liver, with quolls consistently showing higher concentrations than bandicoots (p < 0.001).

### Lung

Quolls showed a significant negative relationship between the concentration of metals in hair and the concentration in the lung only for Cu (ρ = −0.62, p = 0.007; Fig 4). For all remaining metals, no relationship was observed between the concentration in hair and the concentration in the lung (Table 1).

Bandicoots showed no significant relationships between the concentration of metals in hair and the concentration in the lung (Table 2). Quolls and bandicoots showed a significant difference between each other in tissue concentrations for all metals in the lung, with quolls consistently showing higher concentrations than bandicoots (p < 0.001).

### Neocortex

Quolls showed a significant relationship between the concentration of metals in hair and the concentration of metals in the neocortex for Al (ρ = 0.60, p = 0.007; Fig 2), Co (ρ = −0.53, p = 0.02; Fig 4), and Mn (ρ = 0.52, p = 0.02; Fig 2). For all remaining metals, no relationship was observed between the concentration in hair and the concentration in the neocortex (Table 1).

Bandicoots showed no significant relationships between the concentration of metals in hair and the concentration in the neocortex (Table 2). Quolls and bandicoots showed a significant difference between each other in tissue concentrations for all metals in the neocortex, with quolls consistently showing higher concentrations than bandicoots (p < 0.001).

### Olfactory bulb

Quolls showed no significant relationships between the concentration of metals in hair and the concentration in the olfactory bulb (Table 1).

Bandicoots showed a significant negative relationship between the concentration of metals in hair and the concentration in the olfactory bulb for Co (ρ = −0.83, p = 0.008) and Cr (ρ = −0.85, p = 0.006) (Fig 3). For all remaining metals, no relationship was observed between the concentration in hair and the concentration in the olfactory bulb (Table 2). Quolls and bandicoots showed a significant difference between each other in tissue concentrations for all metals in the olfactory bulb, with quolls consistently showing higher concentrations than bandicoots (p < 0.001).

### Testes

Quolls showed no significant relationships between the concentration of metals in hair and the concentration in the testes (Table 1).

Bandicoots also showed no significant relationships between the concentration of metals in hair and the concentration in the testes (Table 2). Quolls and bandicoots showed a significant difference between each other in tissue concentrations for all metals in the testes, with quolls consistently showing higher concentrations than bandicoots (p < 0.001).

## Discussion

Within an animal, different organs often accumulate different amounts of a specific metal (i.e., tissue tropism) [31,32]. For example, following exposure to fine particulate matter, toxic metals preferentially accumulated in different body tissues of rats: Mn accumulated in the brain and liver, Al in the brain, and Cu in the liver [33]. Organs including the liver, kidney, and bone are commonly used to assess enrichment levels of toxic metals in wildlife, providing important information regarding environmental conditions and exposure risks [34]. Metal accumulation in different tissues/organs may vary based on taxonomic group, sex, age, and metal speciation (e.g., methylated vs. elemental Hg). Wildlife that varies in trophic ecology, such as quolls and bandicoots, may be analyzed in tandem as bioindicators of toxic metal pollution to monitor exposure risk, tissue/organ distribution characteristics, and effects on populations. Noninvasive samples (e.g., hair) and samples from euthanized animals (e.g., kidney, liver, muscles) have both been used in biomonitoring studies of environmental metal pollution [34,35]. These studies may be directed towards evaluating health risks for human consumption [36,37] or to understand impacts on wildlife health and reproduction [32].

### Quoll tissue tropism

We previously found that Mn accumulated at higher concentrations in the hair, cerebellum, neocortex, and testes of quolls collected near mining sites than in quolls collected distant from mining sites on Groote Eylandt [8]. We expected to find a significant relationship between the concentration of Mn in hair for both quolls and bandicoots and the organs previously determined to accumulate Mn in quolls (cerebellum, neocortex, and testes). In the current study, we found that hair reflects local exposure, as indicated by proximity to mining operations, of Mn and Al for quolls (Fig 1). We also found that Mn in hair only had a significant positive relationship with Mn in the cerebellum and neocortex, and not with the testes (Fig 2). Our previous study of quolls [8] only analyzed relationships between hair and internal tissues/organs for Mn, and the discrepancy is due to the use of different statistical tests (nonparametric in this study).

Al showed the highest number of significant relationships, with correlations between the concentration of Al in hair and Al in the cerebellum, neocortex, liver and kidney of quolls. Al is the third most abundant element in the Earth’s crust, making it more abundant than Mn [38], including in the dust on Groote Eylandt [8]. The cerebellum, neocortex, and kidney all showed a positive relationship with Al concentration in hair (Fig 2), while Al in the liver showed a negative correlation with Al concentration in hair (Fig 4). Given that we found positive relationships between Al concentration in the hair with concentrations in the cerebellum, neocortex, and kidney, we might expect that brain and kidney accumulate Al. This is supported by the literature, although relative rates of accumulation vary by species. For example, rats exposed to respirable Al accumulated Al in the brain [39,40], while in humans, accumulation of Al after oral exposure was higher in the liver and kidney than in the brain [41,42]. Al concentration increases with age in the kidney and liver [41,42]. Al has no known physiological role but exposure to high levels of Al can impact the musculoskeletal system, kidney, liver, and respiratory and nervous systems via oxidative stress and inflammatory events eventually leading to tissue damage [38,43,44].

In addition to Mn and Al, we found relationships between the concentrations of Co, Cu, and Zn in hair and internal concentrations in tissues/organs for quolls. We found that Co showed a significant positive correlation between the concentration in hair and liver of quolls (Fig 2) but a negative correlation between the hair and neocortex (Fig 4). These results suggest that the liver might be expected to accumulate Co. Rats treated with low concentrations of Co accumulated significant levels of Co in the kidney, liver and brain [45]. Cu showed a significant negative relationship between the concentration in hair and in the lungs of quolls (Fig 4). Excess Cu induces oxidative stress and inflammation leading to tissue damage [46]. Zn showed a significant negative relationship between the concentration in hair and in the kidney and liver of quolls (Fig 4; S1 Fig). These results do not suggest where Zn may accumulate in the body, but Zn accumulates in the kidney of lambs (*Ovis aries*) and cattle (*Bos taurus*) fed a diet high in Zn [47]. Two studies found a negative correlation between age and the concentration of Zn in the liver of striped dolphins (*Stenella coeruleoalba*) and harbor seals (*Phoca vitulina richardii*) [48,49]. In summary, hair appears to be an effective and non-invasive biomonitoring tissue to monitor internal concentrations of Mn and Al in some tissues in quolls (e.g., cerebellum and neocortex), and to some extent also the concentrations of Co, Cu, and Zn.

### Bandicoot tissue tropism

Qualitatively, hair also appears to reflect local exposure of Mn in bandicoots (Fig 1); however, due to our small sample size, more data are necessary to test this relationship.We expected to find comparable relationships between the concentrations of metals in hair and tissues/organs of bandicoots as we did in quolls, and that bandicoots would also show accumulation in the hair, cerebellum, neocortex, and testes based on capture location relative to the mining sites. However, although bandicoots had higher concentrations of some metals in the hair than did quolls (e.g., Al and Mn; Fig 1), we found that bandicoots showed a significantly lower metal concentration in all internal tissues/organs compared to quolls, and that relationships between concentrations in hair and internal tissues/organs were negative rather than positive (Fig 3), whereas quolls exhibited both positive (Fig 2) and negative relationships (Fig 4).

Concentrations of Mn and Al displayed significant negative relationships between hair and the cerebellum in bandicoots (Fig 3). Bandicoots also had a significant negative correlation between concentrations of Co and Cr in hair and in the olfactory bulb (Fig 3). Co and Cr are both associated with tissue damage at high concentrations [50]. Animal and cell culture models demonstrate that Cr accumulates in the brain and induces oxidative damage with widespread neurodegeneration [51]. The brain shows the greatest level of Mn accumulation [7,13,14] and so finding minimal Mn and other respirable metals in the olfactory bulb, cerebellum, and neocortex of bandicoots compared to accumulation in quolls was unexpected.

### Comparisons of quolls and bandicoots

Visible dust containing high levels of Mn covers the substrate in areas surrounding the mine on Groote Eylandt [8], likely exposing quolls and bandicoots living near the mine to higher levels of Mn than those living further away. The average home range of female quolls is 0.35 km^2^ [52]. Male quolls have a similar home range as do females but show an increase during the mating season [52]. Bandicoots have a smaller home range than do quolls, with an average of 0.015 km^2^ [53]. Therefore, for both quolls and bandicoots, the home ranges are sufficiently small to explain differences in Mn content between animals collected near vs. far from the mine (Fig 1).

The dust contains other toxic metals in addition to Mn [8], and therefore impacts of Mn exposure on wildlife are likely exacerbated by exposure to these other metals. We saw large inter-individual variations in metal accumulation in bandicoots (Fig 1) that could be due to individual variation in diet and home range use. Bandicoots are insectivores that spend much of their time digging to obtain food. Most bandicoots collected near mining sites accumulated higher concentrations of Mn in their hair than did quolls (Fig 1), perhaps due to their burrowing behavior. However, their internal tissue concentrations were lower than those observed in quolls. These differences could be due to different rates of molting as the loss of hair is a route of excretion, although rates of molting for the two species are unknown. The differences in life history of bandicoots (digging insectivores [54]) and quolls (semi-arboreal carnivores with a diverse diet that includes vertebrates [18]) and associated physiological parameters such as basal metabolic rate (lower in bandicoots [55]) may explain the dissimilarities in tissue tropism seen in the two species, driven by differences in exposure, uptake, biotransformation, redistribution, and excretion.

Negative correlations observed between metal concentrations in hair and internal tissues in bandicoots may be due to differences in physiology between smaller and larger individuals or based on age. For example, liver Cu concentrations are negatively correlated with body length and/or age in some cetaceans [56,57]. Smaller and younger individuals of suspension feeding bivalves and snails (*Cepaea hortensis*) show higher metal concentrations than do larger and older ones, which could be attributable to their higher metabolic rate [58,59].

Bandicoots showed no obvious trends in the relationship between concentration of metals in tissue and body mass or body length (S1 and S2 Tables). In running 54 tests for body mass (six tissues/organs by nine metals) and 54 tests for body length (also six tissues/organs by nine metals) with an α value of.05, we expected to find approximately three relationships to be significant by chance for each set of tests. Body mass showed significant relationships with the concentration of seven metals (S1 Table), four of which were in the liver. Only three of the correlations were significant for body length (S2 Table), one of which was also in the liver. When we compared the bandicoot results for body mass and body length with the same analyses for quolls (with the addition of kidney data for quolls), we found that body mass showed significant relationships with the concentration of five metals in quolls, four of which were also seen in body length (S3 and S4 Tables). For both body mass and body length in quolls, one of the significant relationships was in the liver, whereas three significant relationships for body mass were in the neocortex and two for body length were also in the neocortex. Together, the quoll and bandicoot data for relationships between tissue/organ concentrations of metals and body mass and body length suggest that the two species differ in their tissue tropism in a way that our data are unable to resolve.

### Conservation implications

Our previous behavioral work on quolls showed that Mn concentration in hair was associated with reduced performance negotiating a turn at speed [22]. This work should be replicated with bandicoots and other wildlife to understand possible impacts of Mn on motor performance in species with different life histories. Conservation measures must account for variation in contaminant exposure due to habitat use and ecological interactions. Insectivores tend to bioaccumulate metals at greater rates than do herbivores because the insects they eat are efficient accumulators themselves [60]. Species that are predatory and/or sedentary are expected to accumulate more contaminants over their lifetimes [61]. Within a species, individuals may spend differing amounts of time in contaminated areas while they feed, mate, or engage in other activities [62–64]. Effective conservation relies on understanding changes in behavior due to exposure to environmental contaminants, such as alterations in feeding, mating, and predator avoidance [65]. Altered behavior (e.g., feeding and home range use) may in turn impact exposure. More generally, exposure to contaminants such as toxic metals can impact many variables relevant to conservation, such as reproductive behavior (impacting birth rates), tissue damage (impacting death rates), and motor function (impacting movement patterns). Focused attention on these parameters for vulnerable species near contaminated sites (such as mines) is warranted and can lead to management solutions to reduce exposures.

## Limitations and conclusions

The small sample size of bandicoots limited the power to detect relationships between metal concentrations in hair and the tissues/organs analyzed. With so many comparisons, we expect some false significance. Nevertheless, our results showed clear differences between bandicoots and quolls in metals accumulation (quolls accumulated higher concentrations than bandicoots in internal tissues/organs while bandicoots accumulated higher concentrations in hair) and in relationships between the concentrations of metals in hair and internal tissues/organs of quolls (positive and negative) and of bandicoots (all negative). These differences likely reflect different physiologies and life histories. Furthermore, we found that the concentration of Mn in the hair of quolls reflected the concentration of Mn in the cerebellum and neocortex, while the concentration of Al in the hair of quolls reflected Al concentration in the cerebellum, neocortex, liver, and kidney (Table 1; Figs 2 and 4). Therefore, quoll hair can be used as a non-invasive biomarker of internal concentration for key tissues such as cerebellum and neocortex for Mn and Al. Groote Eylandt is an area of international conservation significance [16], and hence an improved understanding of the impacts of toxic metals on wildlife should be integrated into conservation strategies. These strategies might include mitigation measures to reduce dust from mining activities, which would benefit both people and wildlife.

## Supporting information

S1 Table The Spearman’s rank correlation coefficient (ρ) and the significance (p) between the concentration of metals in each target tissue and body mass for bandicoots. (DOCX)

S2 Table The Spearman’s rank correlation coefficient (ρ) and the significance (p) between the concentration of metals in each target tissue and body length for bandicoots. (DOCX)

S3 Table The Spearman’s rank correlation coefficient (ρ) and the significance (p) between the concentration of metals in each target tissue and body mass for quolls. (DOCX)

S4 Table The Spearman’s rank correlation coefficient (ρ) and the significance (p) between the concentration of metals in each target tissue and body length for quolls. (DOCX)

S1 Fig Quolls showed a significant negative relationship between the concentration of (A) Co in hair and in the neocortex (ρ = −0.53, p = 0.02), (B) Cu in hair and in the lung (ρ = −0.62, p = 0.007), (C) Zn in hair and in the kidney (ρ = −0.53, p = 0.02), (D) Al in hair and in the liver (ρ = −0.45, p = 0.05), and (E) Zn in hair and in the liver (ρ = −0.53, p = 0.02). These figures show the outliers that were removed in the primary figures.
(TIF)

S1 Data Concentrations of metals in tissues of male northern quolls and northern brown bandicoots sampled on Groote Eylandt, Australia. (XLSX)
