# Supplementary material for: Tissue tropism of toxic metals in northern quolls (Dasyurus hallucatus) and northern brown bandicoots (Isoodon macrourus) on Groote Eylandt, Australia
Source: PLoS One. 2025 Jun 25;20(6):epone.0322386. doi: 10.1371/journal.pone.0322386 (PMC12194021; doi:10.1371/journal.pone.0322386)

**Supplemental Table 4.** The Spearman’s rank correlation coefficient (ρ) and the significance correlation (p) between the concentration of metals in each target tissue and body length for quolls.
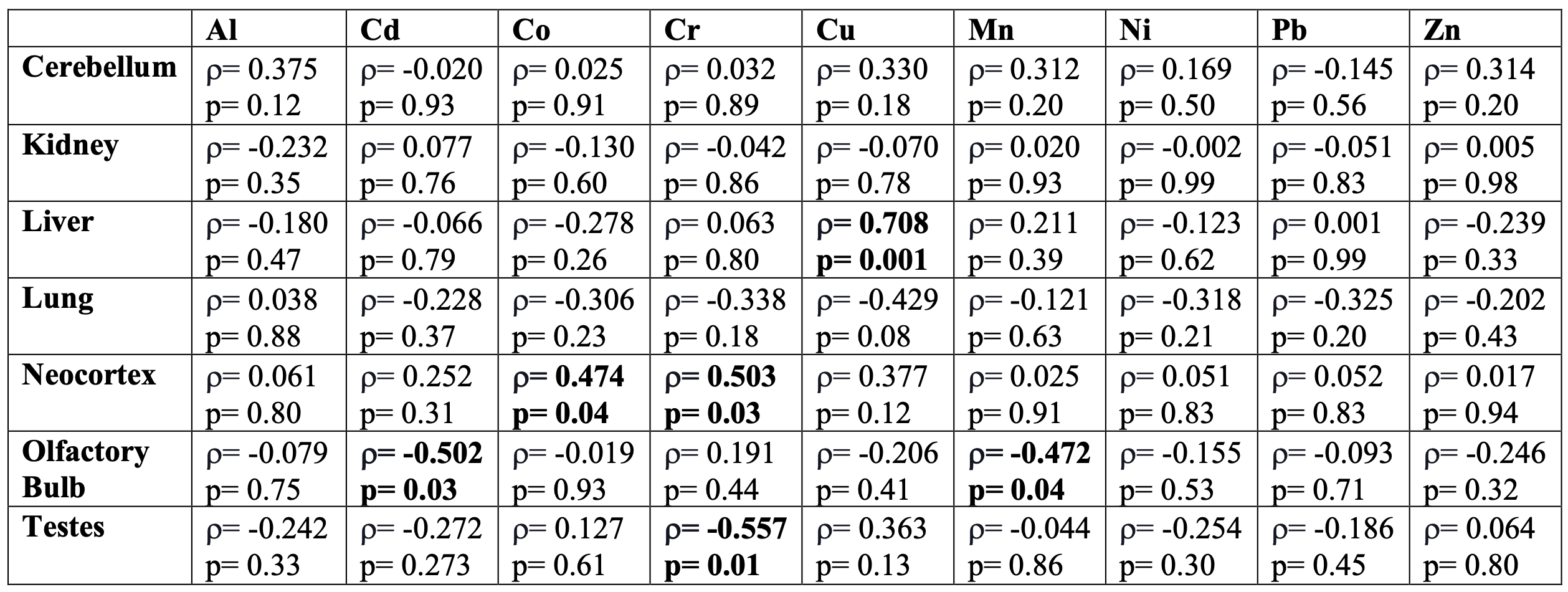

Supplement: S4 Table — (DOCX) [file pone.0322386.s004.docx]
