# Supplementary figures and images for: Tissue tropism of toxic metals in northern quolls (Dasyurus hallucatus) and northern brown bandicoots (Isoodon macrourus) on Groote Eylandt, Australia
Source: PLoS One. 2025 Jun 25;20(6):epone.0322386. doi: 10.1371/journal.pone.0322386 (PMC12194021; doi:10.1371/journal.pone.0322386)

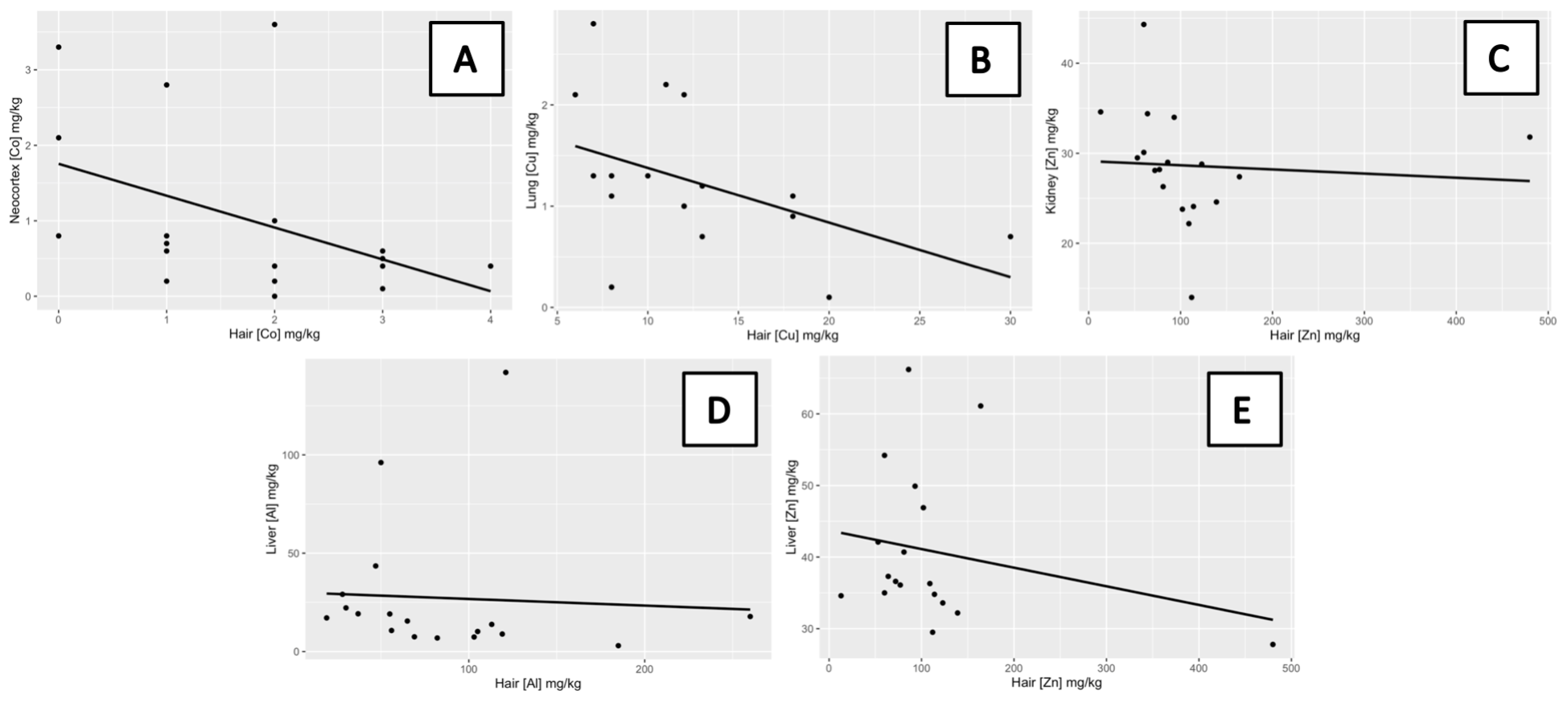

Supplement: S1 Fig — These figures show the outliers that were removed in the primary figures. (TIF) [file pone.0322386.s005.tif]
